# Effects of Tablet Tilt Angle on Hand and Wrist Muscle Activation During Digital Handwriting: A Cross-Sectional Electromyographic Study in University Students

**DOI:** 10.3390/jcm15041514

**Published:** 2026-02-14

**Authors:** Shanyuan Meng, Dong-Kyun Koo, Jung-Won Kwon

**Affiliations:** 1 Department of Physical Therapy, College of Health Sciences, Dankook University, 119, Dandae-ro, Dongnam-gu, Cheonan-si 31116, Chungcheongnam-do, Republic of Korea; mainsy01@dankook.ac.kr; 2 Regional Innovation System & Education Center, Industry-Academic Cooperation Foundation, Wonkwang Health Science University, 514, Iksan-daero, Iksan-si 54538, Jeonbuk-do, Republic of Korea

**Keywords:** electromyography, Ergonomics, hand, wrist, muscle contraction, writing

## Abstract

**Background:** Prolonged tablet use for digital handwriting is increasingly common in educational settings, yet optimal ergonomic positioning remains unclear. This exploratory, cross-sectional study examined how tablet tilt angle affects hand and wrist muscle activation patterns during digital handwriting. **Methods:** This cross-sectional study recruited fifteen healthy university students (age 22.3 ± 2.2 years) who completed standardized writing tasks at three tablet tilt angles (0°, 20°, 60°). Surface electromyography recorded activation from four muscles responsible for the dynamic tripod grip: abductor pollicis brevis (APB), flexor pollicis longus (FPL), flexor digitorum superficialis (FDS), and extensor carpi ulnaris (ECU). **Results:** Significant differences in muscle activation were observed across angles (*p* < 0.05) for three muscles. APB activation was higher at 0° (18.68 ± 11.88% MVIC) and 20° (18.72 ± 12.13% MVIC) than at 60° (14.67 ± 10.38% MVIC), while FDS use decreased from 0° (10.98 ± 4.80% MVIC) to 60° (6.43 ± 3.14% MVIC). Conversely, ECU use increased from 0° (11.76 ± 6.96% MVIC) to 60° (16.15 ± 8.02% MVIC). FPL showed no significant differences. **Conclusions:** Tablet tilt angle substantially affects neuromuscular activation patterns during digital handwriting. In healthy young adults, these findings may help inform preventive ergonomic strategies for prolonged tablet handwriting; however, direct clinical extrapolation requires validation in clinical and more diverse populations.

## 1. Introduction

The rapid advancement of information and communication technologies has significantly altered both daily interactions and professional routines on a global scale. Tablet devices, in particular, have emerged as essential tools in the modern transition to digital engagement. They facilitate a wide range of remote functions, such as virtual learning, teleconferencing, and supporting distributed workforce operations. According to recent data from the Korea Information Society Development Institute (KISDI), tablet penetration reached 31.2% in 2022. This marks a 4.1-percentage-point rise compared to the previous year, indicating a sustained upward trajectory [1]. Parallel improvements in tablet hardware and software—including improved processing capabilities, enhanced display quality, and more responsive user interfaces—have accelerated the movement away from traditional paper-based methods to digital handwriting [2,3].

Handwriting constitutes a complex biomechanical process that relies on the precise coordination of proximal and distal musculoskeletal structures [4,5]. This coordinated motor sequence involves engagement of multiple joint groups. The shoulder and elbow joints play key roles in permitting forearm movement across writing surfaces using a combination of flexion, extension, and rotational actions, while maintaining accurate stylus positioning [6,7]. In tandem, intricate coordination among the digital and carpal joints enables detailed stylus control through regulated flexion and extension motions. The principal motor strategy utilized during handwriting, known as the dynamic tripod grip, engages muscles such as the abductor pollicis brevis, flexor pollicis longus, and flexor digitorum superficialis to achieve fine control [8]. Research by Nadeem and colleagues demonstrated that conventional handwriting is associated with placing the wrist in an extended position with ulnar deviation, with the extensor carpi ulnaris identified as the primary agonist responsible for this movement [9]. Additionally, wrist positioning and support have been shown to significantly affect grip strength and hand function [10].

Most tablets do not incorporate standardized integrated support systems, resulting in substantial differences in the viewing angles chosen by individual users. This design constraint creates notable biomechanical variability, as each user’s posture, joint configuration, and muscle activity are shaped by both the specific case design and the angle at which the device is inclined [11]. Current ergonomic studies indicate that increasing the angle of tablet tilt is associated with lower neck flexion in adults, thereby promoting healthier postural alignment [12]. Additional research has reported increased neck muscle activation when tablets are used on horizontal surfaces, which may contribute to neck discomfort during prolonged use [13]. Further biomechanical evaluation of upper extremity muscle activity during handwriting tasks has shown that a 45° inclination requires the least exertion [2]. Upper extremity posture and position have been demonstrated to significantly influence muscle activation patterns during various tasks [14]. Together, these studies highlight that carefully modifying tablet positioning can substantially affect biomechanical load and has the potential to improve user comfort and ergonomic safety during long sessions of use [15,16].

The biomechanical consequences of varying tablet positioning extend beyond the proximal musculature and exert a notable influence on distal upper extremity function. With increasing tablet inclination, the writing wrist undergoes a progressive extension, approaching the limits of its end range of motion (ROM). Biomechanical research strongly advises against maintaining joints near end-range positions for prolonged periods, given the associated rise in mechanical stress and heightened risk for repetitive stress injuries [17]. This risk is substantiated by Albin and McLoone, whose findings demonstrated peak hand and wrist discomfort at 60° inclinations during writing activities, as evidenced by unfavorable subjective participant reports [18].

Despite numerous studies examining proximal muscle activation in response to various tablet orientations [10,11,12], there remains a substantial gap in knowledge regarding distal upper extremity neuromuscular adaptations. Furthermore, current research has mainly focused on passive forms of tablet interaction (such as reading or simple touch navigation) and has largely overlooked the complex motor control required for digital handwriting [2,19]. Preserving handwriting-related fine motor control and manual dexterity is functionally important in educational contexts (e.g., sustained note-taking) and in therapeutic settings where handwriting may remain a relevant target skill; systematic syntheses of screen-based and technology-assisted writing (including stylus-based tablet writing) further emphasize the need to understand task-specific motor demands [20]. Therefore, the objective of this cross-sectional exploratory study was to quantify hand and wrist muscle activation during controlled tablet handwriting at three tablet inclinations (0°, 20°, and 60°) in university students. It was hypothesized that deliberate changes in tablet positioning would result in significant modifications in hand and wrist muscle activation patterns, reflecting the altered wrist postures associated with each position.

## 2. Materials and Methods

### 2.1. Participants

This cross-sectional study is reported in accordance with the STROBE recommendations for observational studies (cross-sectional design). This cross-sectional investigation enrolled 15 healthy university students (3 males, 12 females; age range: 20–25 years) from a single local institution. The sample size was determined based on a prior exploratory analysis [2], aiming to detect initial neuromuscular adaptation trends. Because the present work was designed as an exploratory/pilot investigation with intensive EMG procedures, the sample size was primarily feasibility-driven and intended to estimate effect magnitudes; therefore, an a priori power calculation was not performed. Effect sizes and confidence intervals are reported to inform the design of future adequately powered studies. Inclusion criteria required the absence of any musculoskeletal conditions involving the hand or wrist within the preceding year, a lack of ongoing wrist pain management, and evidence of typical handwriting capability. Exclusion parameters included any disorders potentially disrupting performance, such as uncorrected visual impairment, neurological dysfunction affecting hand movements, or a record of surgical procedures involving the hands or wrists. Written informed consent was obtained from all participants after a thorough explanation of research objectives, study methodology, and relevant risks, adhering to the Declaration of Helsinki standards. The Institutional Review Board at Dankook University approved the study protocol (DKU 2023-01-017, approved on 5 April 2023). Informed consent was obtained from all individual participants included in the study.

### 2.2. Experimental Apparatus and Materials

#### 2.2.1. Integrated Desk

**Figure 1:**
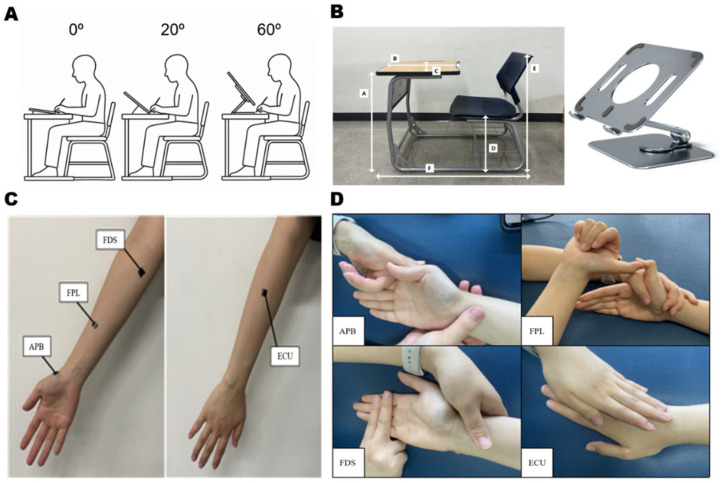
Overview of the experimental design, instrumentation, and electromyographic (EMG) evaluation procedures. (**A**) Schematic diagram indicating the three experimental scenarios, with a subject conducting digital handwriting on a tablet set at 0°, 20°, and 60° inclination. (**B**) The customized integrated desk (Left; Key dimensions: A = 73 cm, B = 47 cm, C = 59.7 cm, D = 41.7 cm, E = 79.2 cm, F = 31.3 cm) and the adjustable tablet stand (Right) that were utilized for data collection. (**C**) Locations of surface EMG electrodes for the abductor pollicis brevis (APB), flexor pollicis longus (FPL), flexor digitorum superficialis (FDS), and extensor carpi ulnaris (ECU) muscles. (**D**) Standardized positions employed during the assessment of maximum voluntary isometric contraction (MVIC) for the targeted muscles, establishing the baseline for EMG signal normalization.

A standardized integrated desk, commonly found in university lecture environments, was selected to authentically replicate academic writing conditions. The exact dimensions of the desk, as measured and shown in Figure 1B, correspond to typical ergonomic standards prevalent in educational settings. Choosing this apparatus increased the study’s ecological validity by modeling the conditions under which students usually perform tablet-based writing activities.

#### 2.2.2. Digital Writing System

Writing tasks were executed using an iPad Air3 tablet (Apple Inc., Cupertino, CA, USA), specified as 25.06 cm × 17.41 cm × 0.61 cm (height × width × thickness) with a mass of 456 g. The device was operated with an Apple Pencil (1st generation), measuring 17.57 cm in length, 0.89 cm in diameter, and weighing 20.7 g. Goodnotes was adopted as the digital writing interface for its established use in academic and professional environments, as well as its ability to closely approximate traditional writing experiences and produce consistent digital outputs for subsequent analysis.

#### 2.2.3. Tablet Positioning Apparatus

An adjustable commercial tablet stand (Figure 1B) was used to accurately orient and secure the tablet at defined angles. This stand consisted of a rectangular base (14.9 cm × 11.8 cm, width × depth) placed on the desk, while its support platform was positioned 13.7 cm above the base. The tablet was inclined to set positions of 0°, 20°, and 60° from horizontal using a calibrated goniometer, which ensured precise and consistent angles throughout all experimental conditions.

#### 2.2.4. Standardized Writing Protocol

**Figure 2:**
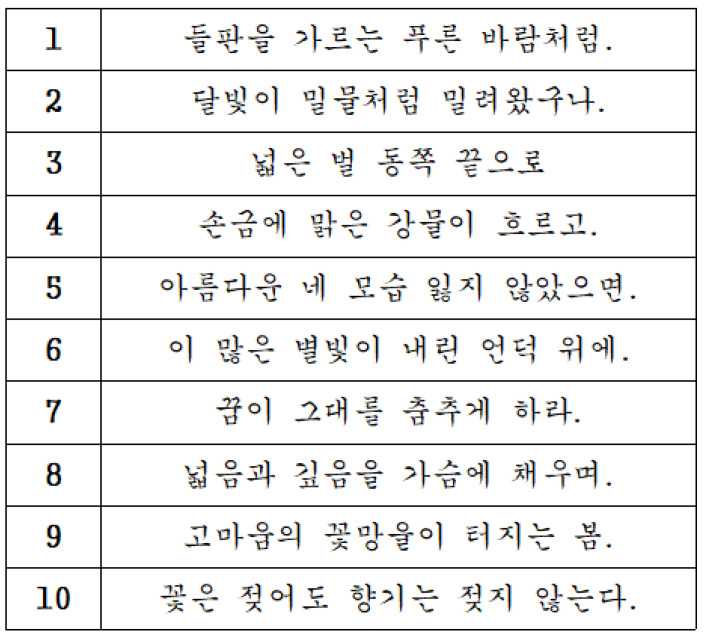
Standardized sentences used as stimuli, extracted from the handwriting verification items of the College Scholastic Ability Test (CSAT) in Korean. These validated samples were chosen to ensure consistent phonological difficulty throughout the experimental sessions. Each trial set consisted of 10 sentences with similar consonant and vowel distributions, minimizing linguistic variability during neuromuscular data collection. (1) Like a verdant breeze sweeping across the fields; (2) The moonlight has surged in like a rising tide; (3) Toward the eastern edge of the vast plains; (4) A clear river flows through the lines of one’s palm; (5) May you never lose your beautiful self; (6) Upon this hill where stars have showered down their light; (7) Let your dreams lead you to dance; (8) Filling the heart with vastness and depth; (9) A spring where gratitude bursts into bloom; (10) Though the flower may be drenched, its fragrance remains undaunted.

The writing protocol consisted of 10 standardized sentences adapted from the handwriting verification section of the College Scholastic Ability Test (CSAT) in South Korea (Figure 2). To limit learning bias and ensure linguistic uniformity, each sentence set was carefully balanced for phonological complexity, with similar distributions of consonants and vowels. Sentences were presented sequentially on a monitor located 5 m in front of the participant. The protocol followed a fixed-random-fixed sequence, in which the initial and final sentences remained unchanged in every session while the middle eight were randomized via a computerized algorithm. Analysis was confined to the eight randomized sentences to minimize the influences of primacy and recency.

#### 2.2.5. Electromyographic Assessment System

Neuromuscular activity was assessed using a Telemyo DTS wireless surface electromyography (sEMG) system (Noraxon USA Inc., Scottsdale, AZ, USA). Signal acquisition parameters were unified with a sampling frequency of 1000 Hz, employing digital bandpass filtering (20–500 Hz) to enhance the signal-to-noise ratio. Skin preparation adhered to recognized protocols for surface electromyography [21], which entailed rigorous cleansing with isopropyl alcohol to decrease impedance and remove surface contaminants. Bipolar Ag/AgCl electrodes were placed parallel to the orientation of muscle fibers, maintaining a 2 cm inter-electrode distance and positioned over the muscle belly to optimize signal detection and mitigate cross-talk (Figure 1C).

Recordings were obtained from four muscles integral to the dynamic tripod grip and wrist stability during handwriting: abductor pollicis brevis (APB), flexor pollicis longus (FPL), flexor digitorum superficialis (FDS), and extensor carpi ulnaris (ECU). These muscles were selected to capture core elements of the dynamic tripod grip and wrist stabilization; however, other wrist extensors and intrinsic hand muscles involved in fine motor control were not assessed. Electromyographic activity was expressed as a percentage of maximum voluntary isometric contraction (%MVIC) to standardize inter-individual comparisons. Maximum voluntary isometric contractions were induced using consistent manual muscle testing techniques (Figure 1D) accompanied by strong verbal encouragement. Each muscle underwent three successive MVIC trials lasting 10 s, with 3 min rest periods between attempts to reduce the risk of fatigue. The mean MVIC value was derived from averaging the central 8 s portion of each trial, excluding the initial and final seconds, to omit non-maximal effort intervals.

### 2.3. Experimental Procedure

Participants were seated in the standardized integrated lecture room chair, following a meticulous posture protocol: feet set at shoulder width, trunk upright, and no contact with the backrest throughout the session. This posture was intended to reduce confounding postural factors influencing upper extremity kinematics. The tablet was customized for each participant to support optimal ergonomic alignment and maintained a consistent orientation across all tested angles, ensuring that tilt angle was the only experimental variable. Surface electromyography electrodes were attached to the dominant upper extremity using the placement guidelines outlined in Section 2.2.5.

The experimental sequence consisted of three distinct segments: familiarization, practice, and data acquisition. In the familiarization phase, participants were thoroughly instructed on experimental tasks and expectations. During the practice phase, participants wrote three sample sentences, not included among the test stimuli, to establish task familiarity and reduce potential learning effects during the measurement trials.

During data acquisition, each stimulus sentence was displayed individually on a monitor, and the tablet interface was set to allow only one sentence per digital writing surface. Each trial commenced following a standardized verbal cue (“start”); participants then transcribed the sentence using their customary handwriting pace and style. Participants were instructed to indicate that the sentence was complete by adding a terminal period. The experimenter, situated on the side of the participant’s dominant hand, observed task progress and advanced the digital interface to the next stimulus upon completion.

The experiment utilized a within-subjects repeated measures design with three distinct tablet inclination conditions (0°, 20°, and 60° from horizontal). The sequence of these conditions was randomized for each participant to address possible order-related confounds. EMG data collection was precisely synchronized to the writing process, beginning at the moment of stylus contact with the tablet and ending when the stylus was lifted following the final period. To minimize fatigue and promote neuromuscular recovery, a fixed 3 min rest interval was scheduled between each angular condition.

### 2.4. Statistical Analysis

All analyses were conducted using IBM SPSS Statistics software (version 26.0, IBM Corporation, Armonk, NY, USA). Descriptive statistics, specifically mean and standard deviation, were reported for all dependent variables. The Shapiro–Wilk test was used to examine data distribution characteristics for muscle activation levels (%MVIC) across each condition. Because the majority of the variables did not satisfy the assumption of normality (Shapiro–Wilk test, *p* < 0.05), non-parametric methods were utilized. The Friedman test was used to evaluate the influence of tablet inclination on muscle activation in the three experimental scenarios (0°, 20°, and 60°), with significance determined at α = 0.05. If significant differences were detected, pairwise comparisons were conducted via the Wilcoxon signed-rank test. To correct for the increased Type I error risk associated with multiple comparisons, all post hoc analyses applied a Bonferroni-adjusted significance level (α = 0.017; 0.05/3 comparisons).

## 3. Results

### 3.1. Participant Characteristics

The sample consisted of 15 healthy university students (3 males, 12 females; age: 22.3 ± 2.2 years; height: 166.5 ± 7.8 cm; weight: 66.2 ± 16.1 kg). The dominant hand assessment revealed 12 right-handed and 3 left-handed individuals. Comprehensive anthropometric and demographic information is provided in Table 1.

### 3.2. Neuromuscular Adaptation Patterns Across Tablet Tilt Angles

**Figure 3:**
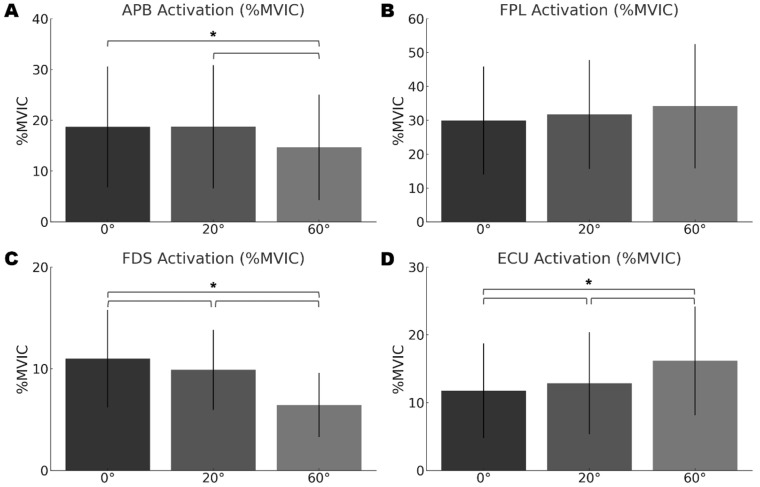
Comparison of muscle activation profiles at varying tablet inclination angles during digital handwriting. Average activation for (**A**) Abductor Pollicis Brevis (APB), (**B**) Flexor Pollicis Longus (FPL), (**C**) Flexor Digitorum Superficialis (FDS), and (**D**) Extensor Carpi Ulnaris (ECU) muscles, represented as a percentage of maximum voluntary isomet-ric contraction (%MVIC). Handwriting was performed at 0°, 20°, and 60° angles. Error bars denote standard deviation (SD). Statistically significant condition differences are denoted with asterisks (*) (*p* < 0.017), as identified by post hoc Wilcoxon signed-rank tests with Bonferroni correction.

Surface electromyography analyses identified distinct patterns of neuromuscular adaptation in the hand and wrist muscles in response to different tablet tilt angles (Table 2, Figure 3). The Friedman test revealed statistically significant differences in muscle activation across three of the four muscles evaluated (*p* < 0.05).

#### 3.2.1. Thenar Musculature

The abductor pollicis brevis (APB) showed significant variation in activation magnitude across the tested tablet inclination angles (χ^2^ = 22.80, *p* < 0.001, Kendall’s W = 0.760), indicating a non-linear response. Bonferroni-adjusted post hoc analysis indicated that APB activation was significantly higher when writing at lower tilt angles (0°: 18.68 ± 11.88% MVIC, 95% CI [12.10, 25.26]; 20°: 18.72 ± 12.13% MVIC, 95% CI [12.00, 25.44]) compared to the 60° position (14.67 ± 10.38% MVIC, 95% CI [8.92, 20.42]) (both comparisons: *p* < 0.017).

Flexor pollicis longus (FPL) activation displayed an incremental increase as tablet inclination rose (0°: 29.93 ± 15.95% MVIC, 95% CI [21.10, 38.76]; 20°: 31.67 ± 16.06% MVIC, 95% CI [22.78, 40.56]; 60°: 34.15 ± 18.35% MVIC, 95% CI [23.99, 44.31]); however, this trend did not reach statistical significance (χ^2^ = 1.73, *p* = 0.420, Kendall’s W = 0.058).

#### 3.2.2. Digital Flexor Musculature

Flexor digitorum superficialis (FDS) activation showed a statistically significant inverse association with tablet inclination (χ^2^ = 17.73, *p* < 0.001, Kendall’s W = 0.591). Step-wise comparisons revealed a gradual decline in FDS activation with increasing tilt, characterized by the highest activation at 0° (10.98 ± 4.80% MVIC, 95% CI [8.32, 13.64]), a moderate value at 20° (9.89 ± 3.94% MVIC, 95% CI [7.71, 12.07]), and the lowest at 60° (6.43 ± 3.14% MVIC, 95% CI [4.69, 8.17]). Post hoc comparisons confirmed statistically significant differences among all inclination levels (all *p* < 0.017).

#### 3.2.3. Wrist Extensor Musculature

Contrary to the digital flexor findings, extensor carpi ulnaris (ECU) revealed a significant direct association with tablet inclination (χ^2^ = 13.73, *p* = 0.001, Kendall’s W = 0.458). ECU activation increased progressively as tablet tilt angle rose, with the lowest activation at 0° (11.76 ± 6.96% MVIC, 95% CI [7.91, 15.61]), a moderate value at 20° (12.86 ± 7.52% MVIC, 95% CI [8.70, 17.02]), and the highest value at 60° (16.15 ± 8.02% MVIC, 95% CI [11.71, 20.59]). All pairwise analyses confirmed significant differences between each condition (all *p* < 0.017), establishing a clear dose–response relationship between tablet angle and ECU activation.

## 4. Discussion

This cross-sectional study investigated distinct neuromuscular adaptation patterns in the hand and wrist muscles of university students across three different tablet inclination angles (0° 20°, and 60°) during digital handwriting tasks. Our results showed significant, angle-specific changes in muscle activation across three functionally defined muscle groups, consistent with evidence that joint positioning influences neuromuscular recruitment strategies [22]. The magnitude of these angle effects was large for APB (Kendall’s W = 0.760) and FDS (W = 0.591) and moderate for ECU (W = 0.458), indicating practically meaningful shifts in neuromuscular demand with tablet inclination. These findings offer empirical support for the biomechanical influence of device orientation on upper extremity muscle activity during the increasingly common practice of digital handwriting in educational contexts, with potential relevance for preserving manual dexterity while reducing preventable musculoskeletal load during prolonged digital note-taking.

### 4.1. Biomechanical Interpretation of Muscle Activation Patterns

#### 4.1.1. Thenar Musculature Response (APB)

The abductor pollicis brevis (APB) demonstrated significantly greater activation at the lower inclination angles (0° and 20°) than at 60°, reflecting a 21.5% decrease in neuromuscular workload at the steepest setting. This result carries notable biomechanical relevance. APB serves as a key stabilizer during the dynamic tripod grip, supporting fine thumb motions through coordinated abduction and opposition [8,23]. The observed pattern can be explained by the length–tension relationship of muscle physiology. At lower inclinations, the wrist usually maintains a neutral or slightly extended position, enabling the thenar muscles to function within their optimal physiological range for force generation [24]. In contrast, the marked decrease in APB activity at 60° likely represents a biomechanical inefficiency caused by excessive wrist extension, possibly stretching the muscle beyond its optimal operational length and thus lowering contractile effectiveness. This is consistent with established evidence that grip force peaks when the wrist is slightly extended [25], which matches the wrist orientation at lower tablet angles. The non-linear APB activation response suggests a threshold: angles above 20° may incrementally impair the efficiency of thenar muscles during precision handwriting tasks.

#### 4.1.2. Neuromuscular Response of FPL and Interpretation of Non-Significance

Conversely, the flexor pollicis longus (FPL) displayed a progressive increase in activation as tablet inclination rose; however, this trend did not reach statistical significance. The absence of statistical significance may be explained by the unique functional role of the FPL in the dynamic tripod grip [8]. Unlike muscles such as the ECU and FDS, which are essential for postural adjustments of the wrist and fingers, the FPL’s primary function is to produce a steady, isostatic force at the interphalangeal joint to stabilize the grip on the stylus [5,23]. It is plausible that the force required for this stabilization remains largely unchanged regardless of wrist angle, since the primary task demand—maintaining controlled pressure on the tablet surface—remains constant. Therefore, although minor variations in FPL activity could occur to accommodate changes in biomechanics at steeper angles, these adjustments did not reach statistical significance in this study [5].

#### 4.1.3. Reciprocal Activity Patterns in Digital Flexors and Wrist Extensors

A notable finding was the contrasting neuromuscular responses of the flexor digitorum superficialis (FDS) and the extensor carpi ulnaris (ECU) to different tilt angles. The Friedman test revealed significant overall differences across angles for both muscles. Further post hoc analyses identified distinct, opposite trends: FDS activation decreased significantly in a stepwise manner as the inclination angle increased from 0°to 20° and again from 20°to 60°, resulting in a total decrease of 41.4% from 0° to 60°. In contrast, ECU activation showed the reverse pattern, rising significantly with each increase in tilt angle (total increase of 37.3% from 0°to 60°). This divergence most likely reflects the biomechanical adaptations required at higher inclinations. Greater wrist extension and ulnar deviation are needed to control the stylus at 60° [9], which inevitably increases the length of the multi-articular FDS [23]. This elongation could augment passive tension in the muscle-tendon unit [26,27], thereby lowering the necessary active contractile force for finger flexion. At the same time, the ECU is required to generate more active force to dynamically stabilize the wrist in this extended and deviated posture, accounting for the observed increase in activation. This observation parallels findings in keyboard research involving wrist posture and extensor muscle activity [28], though the specific task constraints differ. These data demonstrate an adaptive neuromuscular strategy that regulates active force production relative to passive mechanical properties to sustain function under varying postural demands.

### 4.2. Clinical and Educational Implications

The observed biomechanical trade-offs (lower APB load versus higher ECU load at steeper angles) indicate that there is no universally optimal tablet inclination angle for all users; ergonomic recommendations should be individualized to the user and task [13,14]. Increased angles may benefit those with thumb-related concerns by reducing APB load, but may simultaneously exacerbate issues in the wrist extensors due to higher ECU demand, particularly during prolonged periods of note-taking. Providing adjustable stands and brief guidance on these trade-offs may help support musculoskeletal health in educational and clinical settings.

### 4.3. Limitations and Future Directions

Interpretation of these results should take into account several notable limitations. The restricted sample—consisting of a small group of healthy, young university students—reduces the applicability of the findings to broader demographic groups or clinical cohorts. Individual factors such as writing style, which were not controlled for, may contribute additional variability to the results. Additionally, although a familiarization and brief practice phase was included, participants’ prior familiarity with tablet-based handwriting and stylus use was not quantified and could have influenced neuromuscular recruitment; future studies should document prior experience and incorporate longer habituation where appropriate. In addition, EMG recordings were limited to four muscles; therefore, activation of other wrist extensors and intrinsic hand muscles potentially involved in handwriting was not captured. Hand dominance was mixed (12 right-handed, 3 left-handed), and the small number of left-handed participants precluded meaningful stratified analyses; future studies should test whether handedness-related writing strategies influence activation patterns. Moreover, the absence of kinematic, kinetic, and subjective outcome measures precludes construction of a comprehensive biomechanical model and constrains inferences regarding movement strategies and the broader perceptual context of tablet use.

Future investigations should focus on mitigating these limitations. Extended longitudinal studies are necessary to evaluate user adaptation and the development of fatigue over time, an issue of high relevance for students with frequent tablet use. Coupling EMG data with motion capture, force or pressure measurements, and subjective ratings in multimodal protocols will expand our biomechanical insight. Studies recruiting more heterogeneous samples, such as pediatric cohorts or patients with musculoskeletal conditions, would improve the clinical and educational applicability of the results. Future protocols should also incorporate a broader set of upper-extremity and intrinsic hand muscles and be designed to allow stratification by handedness. Additionally, research exploring how tablet inclination interacts with other ergonomic parameters will facilitate development of more comprehensive guidelines.

## 5. Conclusions

This research establishes that tablet inclination angle has a substantial effect on neuromuscular activation patterns in the hand and wrist during digital handwriting. A steeper tablet orientation results in marked reductions in abductor pollicis brevis demands but increases extensor carpi ulnaris activation, along with decreased finger flexor (FDS) engagement. These variable neuromuscular responses underscore the influence of postural adaptation and muscle mechanics. The current evidence effectively supports the need for more refined ergonomic recommendations tailored to the specific demands of digital handwriting tasks. Adapting tablet configuration according to individual needs and activity context may improve user comfort, decrease fatigue risk, and foster musculoskeletal wellbeing among students in technology-driven educational settings. These findings may also support preventive strategies aimed at maintaining manual dexterity and efficient handwriting-related motor control in digital learning environments; however, validation in broader and clinical populations is needed before direct clinical recommendations are made.

## Figures and Tables

**Table 1:** Demographic and anthropometric data of study participants (*n* = 15).

Characteristic	Value
Writing hand dominance (L/R)	3/12
Age (years)	22.3 (2.2)
Height (cm)	166.5 (7.8)
Body mass (kg)	66.2 (16.1)

Values are expressed as frequencies or mean (±standard deviation).

**Table 2:** Muscle activation characteristics during digital handwriting at three tablet inclination angles (*n* = 15).

Muscles	Tablet Tilt	Mean (SD) [%MVIC]	95% CI [%MVIC]	χ^2^	Effect Size (Kendall’s W)	*p*-Value	Post Hoc
APB	0°	18.68 (11.88)	[12.10, 25.26]	22.80	0.760	* <0.001	A, B > C
	20°	18.72 (12.13)	[12.00, 25.44]				
	60°	14.67 (10.38)	[8.92, 20.42]				
FPL	0°	29.93 (15.95)	[21.10, 38.76]	1.73	0.058	0.420	-
	20°	31.67 (16.06)	[22.78, 40.56]				
	60°	34.15 (18.35)	[23.99, 44.31]				
FDS	0°	10.98 (4.80)	[8.32, 13.64]	17.73	0.591	* <0.001	A > B > C
	20°	9.89 (3.94)	[7.71, 12.07]				
	60°	6.43 (3.14)	[4.69, 8.17]				
ECU	0°	11.76 (6.96)	[7.91, 15.61]	13.73	0.458	* 0.001	A < B < C
	20°	12.86 (7.52)	[8.70, 17.02]				
	60°	16.15 (8.02)	[11.71, 20.59]				

%MVIC: Percentage of maximum voluntary isometric contraction; SD: Standard Deviation; APB: Abductor pollicis brevis; FPL: Flexor pollicis longus; FDS: Flexor digitorum superficialis; ECU: Extensor carpi ulnaris. A, B, and C denote tablet inclination angles of 0°, 20°, and 60°, respectively. *: Indicates a statistically significant difference for the main effect (Friedman test, *p* < 0.05). Post hoc comparisons in the final column were conducted using the Wilcoxon signed-rank test with Bonferroni correction (adjusted significance threshold *p* < 0.017), identifying significant differences between specific angle conditions.

## Data Availability

The data presented in this study are available on request from the corresponding author. The data are not publicly available due to privacy restrictions.

## Abbreviations

The following abbreviations are used in this manuscript:

MVIC	Maximum Voluntary Isometric Contraction
EMG	Electromyography
APB	Abductor Pollicis Brevis
FPL	Flexor Pollicis Longus
FDS	Flexor Digitorum Superficialis
ECU	Extensor Carpi Ulnaris
